# Chylous effusion presenting in a 37-year-old woman with severe hypothyroidism: a case report

**DOI:** 10.1186/1752-1947-4-336

**Published:** 2010-10-25

**Authors:** Kevin SH Koo, Rand Barnard, Frank T Kagawa, Weichia Chen, Irma Hinojosa

**Affiliations:** 1 University of California San Francisco, Department of Radiology and Biomedical Imaging, San Francisco, California 94122, USA; 2 Department of Medicine, Santa Clara Valley Medical Center, 751 South Bascom Avenue, San Jose, CA 95128, USA; 3 Division of Respiratory and Critical Care Medicine, Santa Clara Valley Medical Center, 5th floor Valley Specialty Building, 751 South Bascom Avenue, San Jose, CA 95128, USA; 4 Division of Pulmonary and Critical Care Medicine, Stanford University School of Medicine, 300 Pasteur Drive, Stanford, California 94305, USA

## Abstract

**Introduction:**

We report what is to the best of our knowledge the second adult case of chylothorax clearly associated with severe hypothyroidism in the English-language medical literature. To the best of our knowledge, this is the first case of its kind reported without a prior history of malignancy.

**Case presentation:**

A 37-year-old Hispanic woman with no reported significant past medical history initially presented with shortness of breath and inability to lose weight. She was found to have a large chylous effusion requiring chest-tube drainage, as well as severe hypothyroidism. After several weeks of thyroid hormone-replacement therapy, the formation of chylous pleural fluid in the patient greatly diminished, and the chest tube was removed. Upon long-term follow-up her minimal residual effusion remains stable on serial chest radiographs.

**Conclusion:**

Although the exact pathophysiologic relation between low thyroid hormone levels and chyle formation remains to be elucidated, hypothyroidism should be a diagnostic consideration in patients with chylous effusions, especially those refractory to conventional treatments.

## Introduction

Hypothyroidism is a relatively common disorder among the general population, and its incidence only increases with age. Typically patients present with a subclinical picture, but left untreated, the condition can develop into overt hypothyroidism. The prevalence of overt hypothyroidism has been estimated to be 0.3%, whereas subclinical hypothyroidism can be as high as 4.3% [1].

One previous case report described a chylothorax clearly associated with hypothyroidism [2], however, the patient described in that case report had a history of lymphoma previously treated with radiation therapy to the mediastinum. Here we report what we believe to be the first case of a chylous effusion associated with hypothyroidism in a woman without a prior history of malignancy.

## Case Presentation

A 37-year-old Hispanic woman without a significant past medical history presented to our hospital with a one week history of shortness of breath, which had acutely worsened over the past few days. She stated that she required two pillows to breathe while lying down. She also stated that she could no longer work at her childrens' day care center, as even walking at a relaxed pace for more than two minutes would cause her to be short of breath, although the shortness of breath would improve with rest. She also complained of a nonproductive cough for two days before admission. She denied any trauma, chest pain, palpitations, nausea and vomiting, diarrhea, abdominal pain, or fevers. Review of systems was remarkable for a one month history of bilateral lower extremity edema.

She denied any significant past medical history and had not been taking any medication before hospital admission. She remotely recalled that her last physician, whom she had not seen in the previous eight years, had mentioned to her that she might have hypothyroidism, but this was not further investigated. She denied use of tobacco, alcohol, or any other recreational drugs.

Physical examination revealed an obese, pale woman in mild distress from shortness of breath, but able to speak in full sentences. Her vital signs on arrival showed an oral temperature of 36.8°C, heart rate in the range of 81 to 93 beats per minute, blood pressures ranging from 140 to 173 mm Hg systolic and 94 to 121 mm Hg diastolic. Her breathing was 18 to 22 times per minute, with a digital pulse oximetry saturation of 86% while breathing air, which subsequently improved to 92% when she was given oxygen therapy via a face mask. Her neck examination showed no nodular thyroid or thyroid masses. There was no lymphadenopathy. Dullness to percussion was found over the lower portion of her left hemithorax, accompanied by decreased vocal fremitus, as well as decreased breath sounds on auscultation. The chest examination was normal over her right hemithorax. Cardiac examination revealed no murmurs, rubs, or gallops, but heart sounds were generally distant. Abdominal examination was unremarkable, with no signs of ascites or masses. Lower extremities revealed 2+ pitting edema to the level of the knees bilaterally.

**Figure 1:**
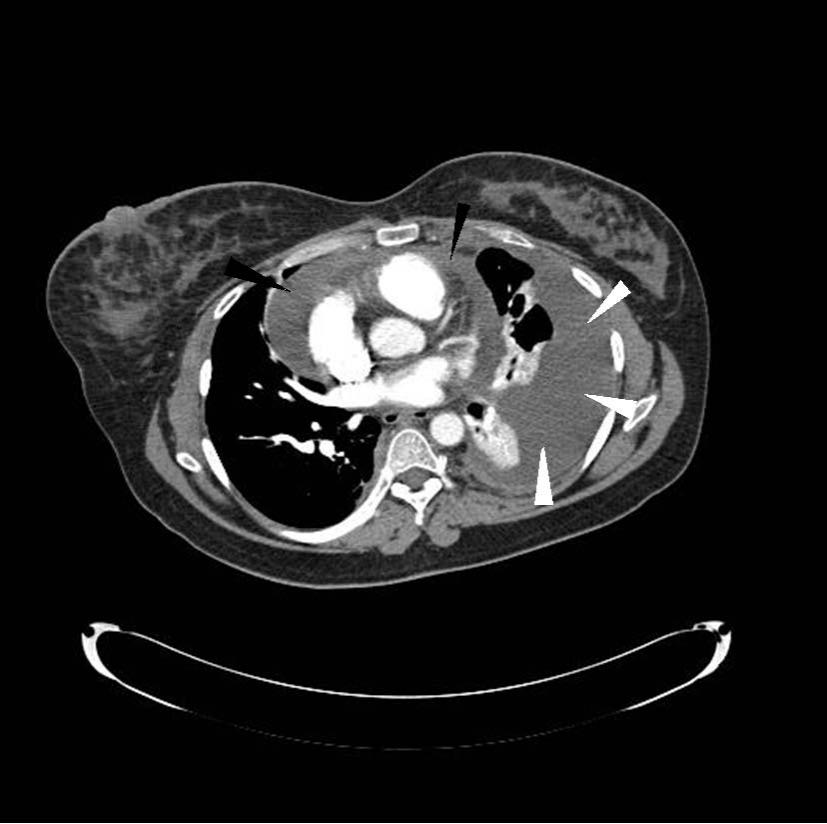
**Computed tomography angiogram of the chest**. A CT angiogram of the chest performed on hospital day one shows a large pleural fluid accumulation in the left pleural space (white arrowheads) with compressive atelectasis of the left lung as well as a pericardial effusion (black arrowheads). No pulmonary embolus was seen. The pleural fluid was later evacuated and found to be chylous.

Her electrocardiogram showed low voltage throughout all leads with no other ST-segment or T-wave abnormalities. A chest radiograph showed an enlarged cardiac silhouette and bilateral pleural effusions, with more fluid on the left than on the right. A computed tomography (CT) angiogram of the chest did not reveal pulmonary thromboembolism and confirmed the presence of a large left pleural effusion with associated compressive atelectasis, as well as a moderate pericardial effusion (Figure 1).

Admission laboratory values demonstrated a leukocyte count of 6.3 × 10^9^/L (normal, 3.5 to 11.0 × 10^9^/L) with a hematocrit of 36% (normal, 36% to 46%). A chemistry panel was remarkable for a creatinine of 1.4 mg/dL (124 μmol/L; normal, 44 to 106 μmol/L). Troponin T cardiac enzyme was slightly elevated at 0.02 μg/L (normal, <0.01 μg/L), and beta-natriuretic peptide was elevated at 1325 ng/L (normal, 0 to 450 ng/L). Urine alaysis was significant for 2+ protein. A fasting lipid panel showed a total cholesterol of 179 mg/dL (4.64 mmol/L; normal, 3.65 to 5.15 mmol/L); triglycerides, 186 mg/dL (2.10 mmol/L; normal, <1.70 mmol/L), HDL, 25 mg/dL (0.65 mmol/L; normal: >1.01 mmol/L), and an LDL of 117 mg/dL (3.03 mmol/L; normal, 1.55 to 3.34 mmol/L). The liver panel showed a total protein of 7.0 g/dL (70 g/L; normal, 60 to 80 g/L) and albumin at 3.3 g/dL (33 g/L; normal, 35 to 50 g/L) with normal transaminases. Lactate dehydrogenase (LDH) was 302 U/L (normal, 112 to 220 U/L). Given her complaints of fatigue, a check of thyroid-stimulating hormone (TSH) and a free T_4 _levels were also carried out. The results of those tests showed the her TSH to be 181.90 mIU/L (normal, 0.27 to 4.2 mIU/L) and her free T_4 _to be <0.1 ng/dL (<1.29 pmol/L; normal, 0.9 to 1.7 pmol/L).

**Figure 2:**
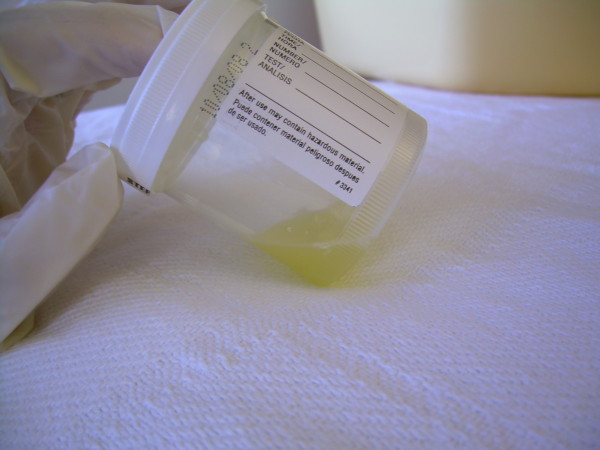
**Chylous pleural fluid sample**. This sample was obtained from the her left chest tube on hospital day 11. Note atypical yellow color of this chylous effusion.

Thoracentesis on hospital day two revealed cloudy yellow fluid (Figure 2) with 129 × 10^9^/L white blood cells (23% neutrophils, 23% lymphocytes, 54% monocytes); a lactate dehydrogenase (LDH) level of 170 U/L, with a pleural fluid LDH-to-serum ratio of 0.56; a protein level of 5.6 g/dL (56 g/L), with a pleural fluid protein-to-serum ratio of 0.8; albumin of 2.8 g/dL (28 g/L); cholesterol of 81 mg/dL (2.10 mmol/L), with a pleural fluid cholesterol-to-serum ratio of 0.5; and triglycerides of 442 mg/dL (4.99 mmol/L), with a pleural fluid triglycerides-to-serum ratio of 2.4. These findings were thought to be compatible with an exudative chylous effusion. A 10-French chest tube was placed by our interventional radiologists the fourth day of her hospitalization. A follow-up chest CT after drainage of the effusion performed the seventh day of hospitalization showed a nonspecific retrocrural density that our radiologists thought to be compatible with swelling or inflammation related to her myxedema.

To investigate the etiology of her chylous effusion, she underwent nuclear medicine lymphatic scintigraphy on the following day to look for possible thoracic duct injury. The study showed normal tracer uptake throughout the lymphatic system without any evidence of accumulation to suggest leakage or trauma.

**Figure 3:**
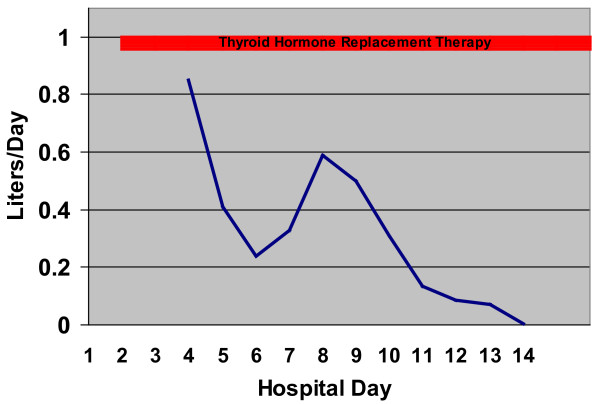
**Graph of chest tube daily output**. This shows the daily output in liters from the her left chylous pleural effusion. The red bar represents the period of thyroid hormone-replacement therapy.

She was prescribed levothyroxine, 100 μg, slowly escalating to 150 μg orally per day for treatment of her hypothyroidism, along with a low-fat diet, and over the course of two weeks, her chest-tube drainage progressively decreased (Figure 3), and her fatigue and dyspnea subjectively improved. Her antithyroid peroxidase antibody was found to be elevated at 97.1 kIU/L (normal, <40 kIU/L), compatible with autoimmune thyroiditis. The chest tube was removed after ten days, and she was discharged home.

At two and three month follow-up visits, her fatigue had significantly improved, and she had returned to regular employment. Her TSH achieved a normal level (1.35 mIU/L; normal, 0.27 to 4.2 mIU/L). Follow-up chest radiographs and CT studies at two, three and six months showed near-complete resolution of the left chylous effusion, with complete resolution of the mild pericardial effusion. The retrocrural density seen on earlier CT studies has remained unchanged. At one year follow-up, no recurrence of her pleural effusion or clinical signs of malignancy have occurred.

## Discussion

Chylothoraces and chylous pleural effusions are a rather rare entity within the adult population. In a case series of 809 patients undergoing thoracentesis at a tertiary medical center, only three percent were found to have a chylothorax [3]. Typically, an effusion is determined to be chylous either by the presence of chylomicrons on lipoprotein analysis, or, as in our case report, by elevated effusion pleural fluid triglycerides greater than 110 mg/dL (1.24 mmol/L) [4]. Because both pseudochylothorax/chyliform effusions and patients with high serum triglycerides can also have elevated pleural fluid triglycerides, additional biochemical criteria are often used to exclude these two conditions, as was done in the present case, including a ratio of pleural fluid to serum triglycerides more than 1.0 and a ratio of the pleural fluid to serum cholesterol level less than 1.0 [5]. From a collection of several series of patients totaling 384 with chylothoraces, Agrawal and Sahn [6] found that the most common etiology was surgical trauma, at 38%. This was followed by tumor (32%), miscellaneous, including sarcoidosis, cirrhosis, congestive heart failure, tuberculosis, and amyloidosis (18%), idiopathic (10%), and nonsurgical trauma (2%) [6]. Of malignancies that can cause a chylothorax, lymphoma is the predominant etiology, accounting for 75% of all malignancies that can cause a chylous effusion, usually as a result of obstruction and subsequent rupture at the thoracic duct or other part of the lymphatic system [6].

To the best of our knowledge, only one prior case has been published in the English language medical literature: an adult patient with a chylous effusion clearly associated with hypothyroidism. In that case, Kollef [2] described a 37-year-old woman with refractory chylous pleural effusions and chylous ascites that resolved only after thyroid hormone-replacement therapy [2]. The patient, however, also had a confounding history of Hodgkin lymphoma (a well-described cause of chylous effusions) that had been treated with thoracic mantle and periaortic radiation, which may have predisposed her to the development of a chylous effusion [5,6].

The pathophysiologic relation between chylothoraces and acquired hypothyroidism in adults continues to be undefined. It has been presumed that the ability of the thyroid hormone to control lipid metabolism [7] may directly affect the development of chyle [2]. This mechanism fails to explain why our case of a massive effusion with high pleural triglycerides should have only minimally abnormal serum triglycerides. An alternative mechanism proposed by Kessel and colleagues [8] for infants born with congenital hypothyroidism and chylous effusions from non-immune hydrops fetalis might also explain chyle formation in adult patients with acquired hypothyroidism. In their "reduced adrenergic stimulation hypothesis," thyroid hormone may play a role in the regulation of adrenergic receptors in the lymphatic system and lungs, thus modulating both the lymphatic flow rate and lung liquid clearance and facilitating the formation and resolution of chylothorax [8].

We performed an extensive search for alternative etiologies for our her chylothorax. To rule out thoracic duct injury, we performed a lymph node scintigraphy scan, which was normal. We performed CT scans to look for evidence of cancer or lymphoma, which, with the exception of nonspecific findings in the retrocrural area suggestive of inflammation, were unremarkable. Furthermore, no signs were suggestive of malignancy at one year of clinical follow-up. No other signs suggested that any of the other etiologies discussed caused her chylous effusion to develop, other than occult severe hypothyroidism. Although we still cannot exclude the possibility that this was a coincidental spontaneous chylothorax unrelated to the severe hypothyroidism, the effusion did respond dramatically to thyroid hormone-replacement therapy in a two week time course that was remarkably similar to the case of chylothorax associated with hypothyroidism reported by Kolef [2].

## Conclusion

We report the first case of chylothorax associated with hypothyroidism responsive to thyroid-replacement therapy, without a history of prior malignancy. Although the exact pathophysiologic relation between chylothoraces and hypothyroidism remains to be elucidated, it is important for clinicians to recognize that hypothyroidism maybe a potentially reversible cause of a chylous pleural effusion.

## Patient's Perspective

The experience was scary. My first worry was, "Am I going to die?" When I found out that it was not going to kill me, I was in the clouds. I was so happy to find out that it was my thyroid and not cancer.

## Consent

Written informed consent was obtained from the patient for publication of this case report and accompanying images. A copy of the written consent is available for review by the journal's Editor-in-Chief.

## Competing interests

The authors declare that they have no competing interests.

## Authors' contributions

KSHK, RB, FTK, and WC were all involved in the care of this patient and writing of the manuscript. KSHK wrote the draft manuscript as well as performed the literature searches. RB analyzed the patient's thyroid data and helped with editing of this manuscript. FTK edited the manuscript and analyzed the patient's pleural effusion laboratory data. WC photographed the effusion and edited the manuscript. IH wrote the patient's perspective. All authors have read and approved the final manuscript. FTK had full access to all the data in the study and takes responsibility for the integrity and the accuracy of the data.
